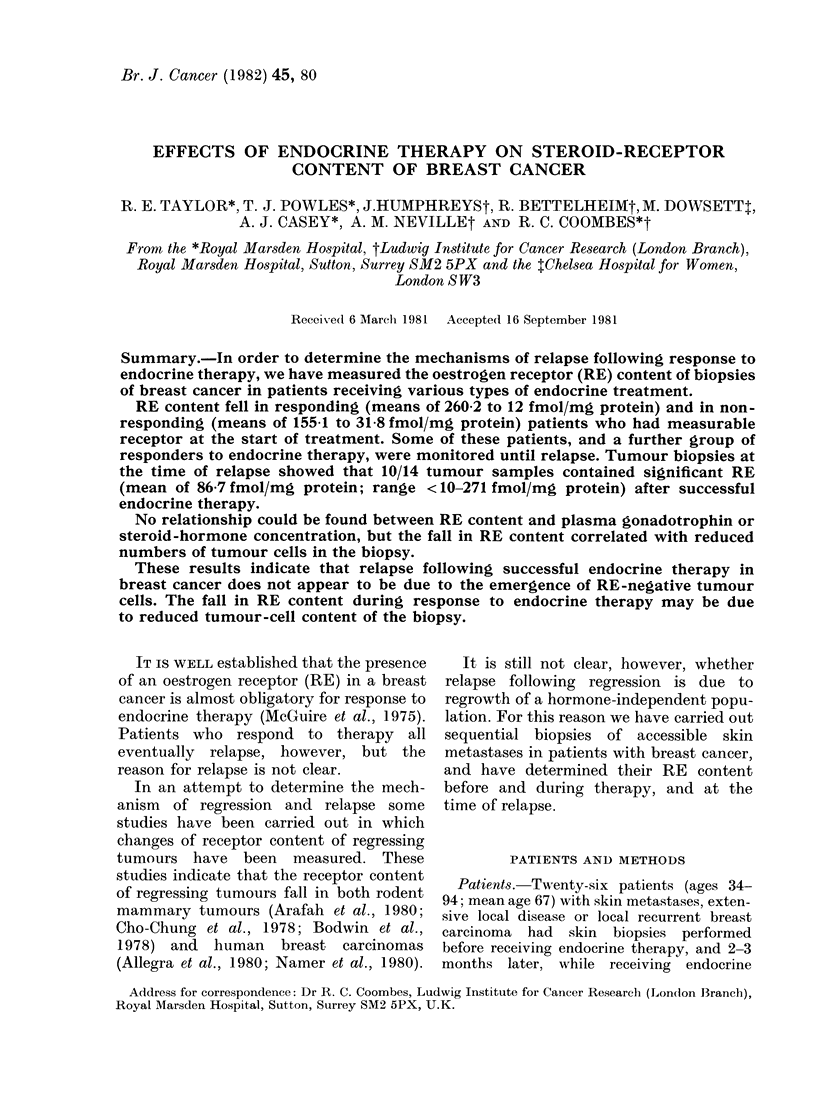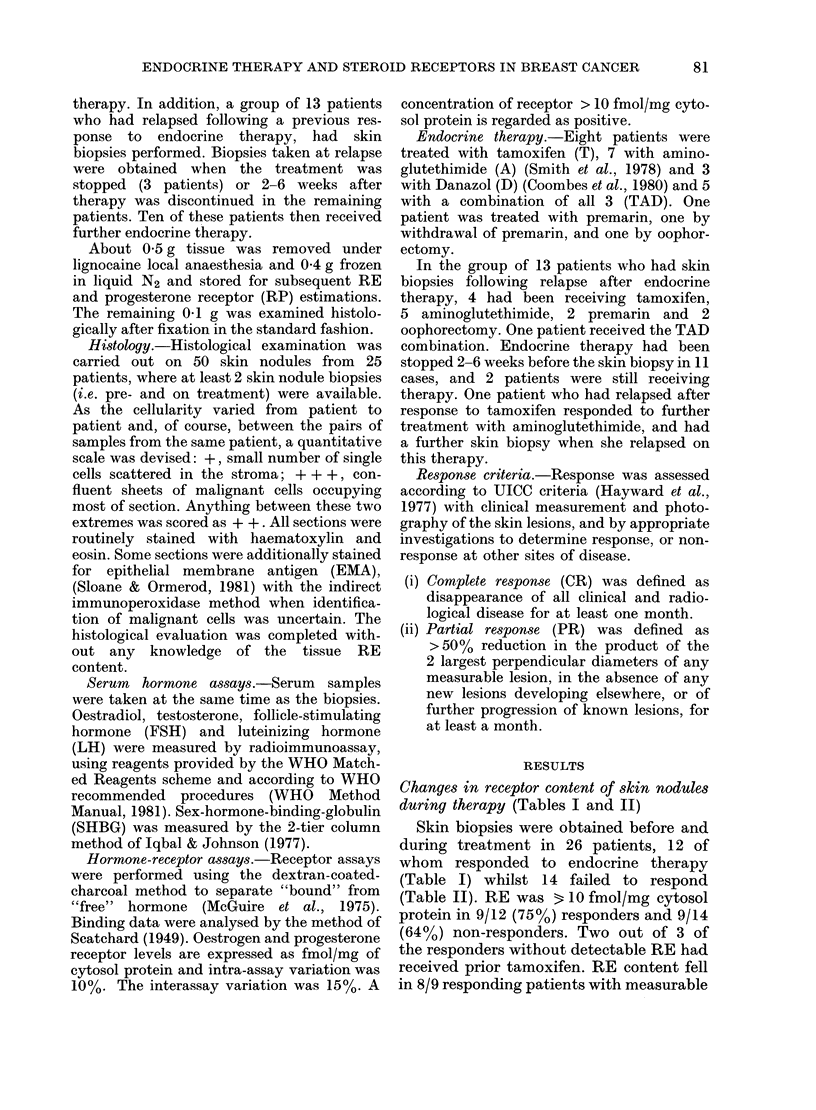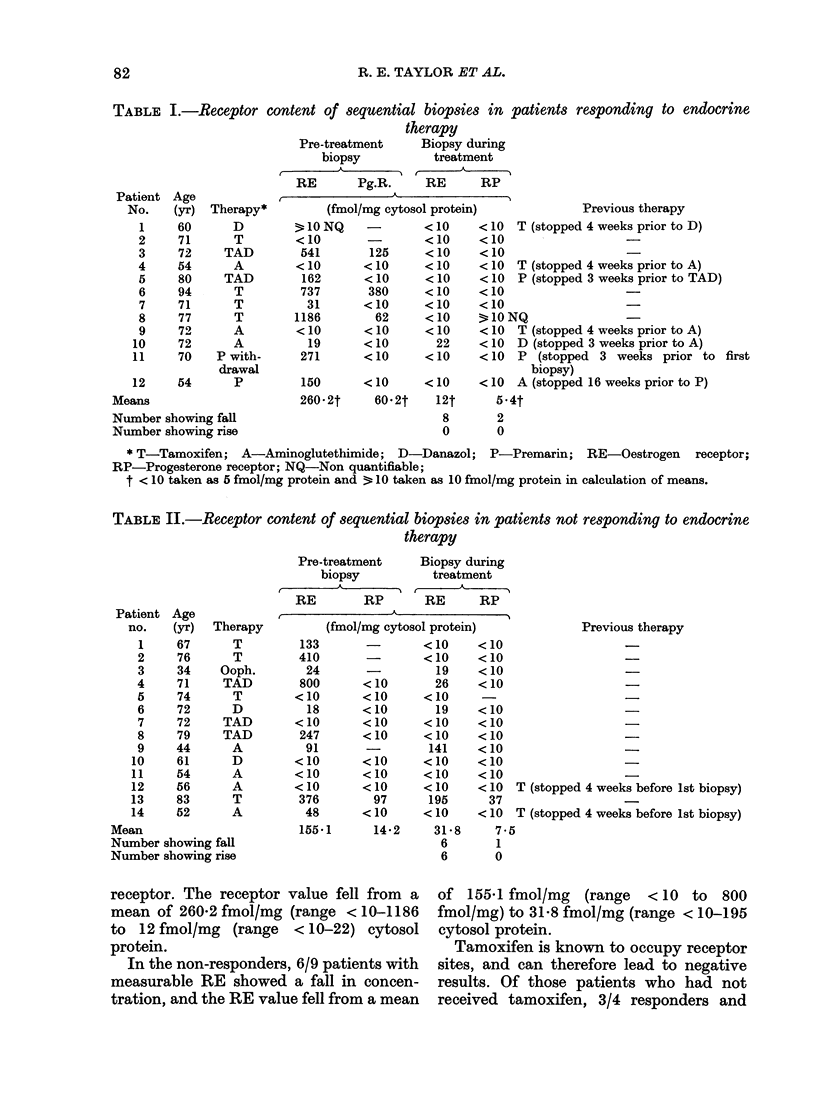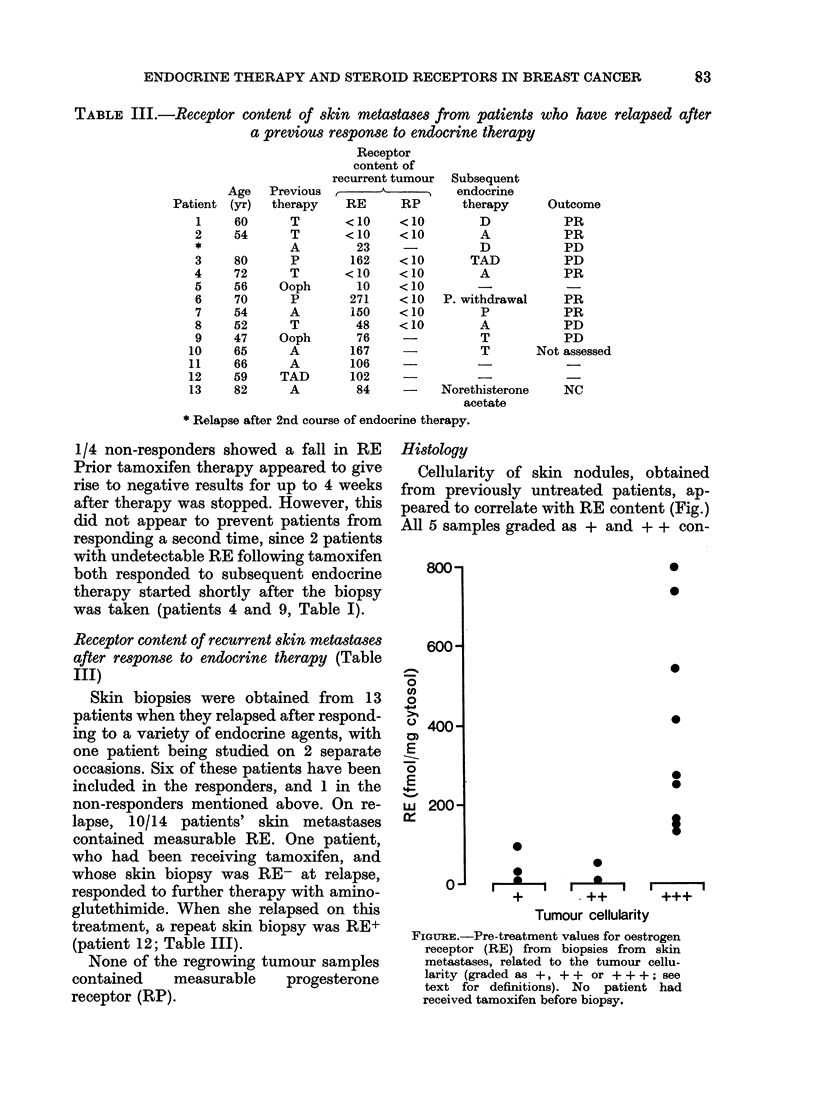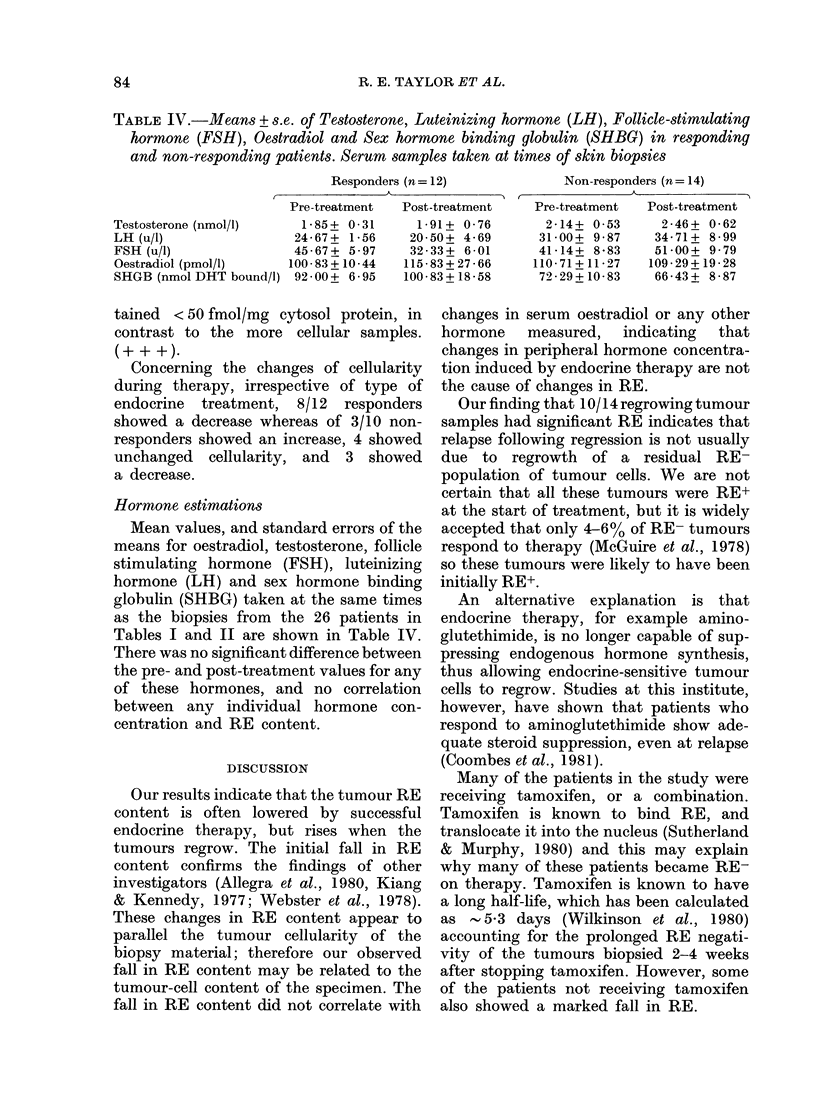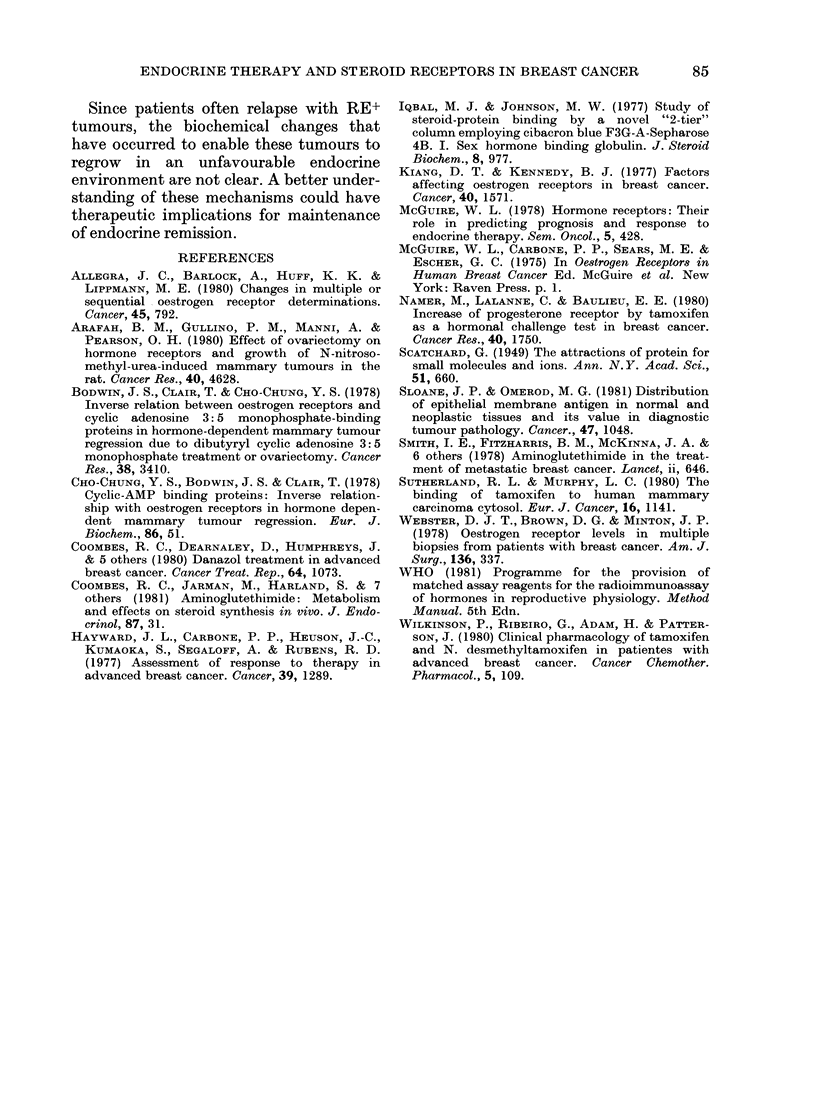# Effects of endocrine therapy on steroid-receptor content of breast cancer.

**DOI:** 10.1038/bjc.1982.10

**Published:** 1982-01

**Authors:** R. E. Taylor, T. J. Powles, J. Humphreys, R. Bettelheim, M. Dowsett, A. J. Casey, A. M. Neville, R. C. Coombes

## Abstract

In order to determine the mechanisms of relapse following response to endocrine therapy, we have measured the oestrogen receptor (RE) content of biopsies of breast cancer in patients receiving various types of endocrine treatment. RE content fell in responding (means of 260.2 to 12 fmol/mg protein) and in nonresponding (means of 155.1 to 31.8 fmol/mg protein) patients who had measurable receptor at the start of treatment. Some of these patients, and a further group of responders to endocrine therapy, were monitored until relapse. Tumour biopsies at the time of relapse showed that 10/14 tumour samples contained significant RE (mean of 86.7 fmol/mg protein; range less than 10-271 fmol/mg protein) after successful endocrine therapy. No relationship could be found between RE content and plasma gonadotrophin or steroid-hormone concentration, but the fall in RE content correlated with reduced numbers of tumour cells in the biopsy. These results indicate that relapse following successful endocrine therapy in breast cancer does not appear to be due to the emergence of RE-negative tumour cells. The fall in RE content during response to endocrine therapy may be due to reduced tumour-cell content of the biopsy.


*Br. J. Cancer* (1982) **45**, 80

### EFFECTS OF ENDOCRINE THERAPY ON STEROID-RECEPTOR CONTENT OF BREAST CANCER

R. E. TAYLOR*, T. J. POWLES*, J. HUMPHREYS†, R. BETTELHEIM†, M. DOWSETT‡, A. J. CASEY*, A. M. NEVILLE† AND R. C. COOMBES*†

*From the *Royal Marsden Hospital, †Ludwig Institute for Cancer Research (London Branch), Royal Marsden Hospital, Sutton, Surrey SM2 5PX and the ‡Chelsea Hospital for Women, London SW3*

Received 6 March 1981 Accepted 16 September 1981

**Summary.—In order to determine the mechanisms of relapse following response to endocrine therapy, we have measured the oestrogen receptor (RE) content of biopsies of breast cancer in patients receiving various types of endocrine treatment.**

**RE content fell in responding (means of 260·2 to 12 fmol/mg protein) and in non-responding (means of 155·1 to 31·8 fmol/mg protein) patients who had measurable receptor at the start of treatment. Some of these patients, and a further group of responders to endocrine therapy, were monitored until relapse. Tumour biopsies at the time of relapse showed that 10/14 tumour samples contained significant RE (mean of 86·7 fmol/mg protein; range <10–271 fmol/mg protein) after successful endocrine therapy.**

**No relationship could be found between RE content and plasma gonadotrophin or steroid-hormone concentration, but the fall in RE content correlated with reduced numbers of tumour cells in the biopsy.**

**These results indicate that relapse following successful endocrine therapy in breast cancer does not appear to be due to the emergence of RE-negative tumour cells. The fall in RE content during response to endocrine therapy may be due to reduced tumour-cell content of the biopsy.**

IT IS WELL established that the presence of an oestrogen receptor (RE) in a breast cancer is almost obligatory for response to endocrine therapy (McGuire *et al.*, 1975). Patients who respond to therapy all eventually relapse, however, but the reason for relapse is not clear.

In an attempt to determine the mechanism of regression and relapse some studies have been carried out in which changes of receptor content of regressing tumours have been measured. These studies indicate that the receptor content of regressing tumours fall in both rodent mammary tumours (Arafah *et al.*, 1980; Cho-Chung *et al.*, 1978; Bodwin *et al.*, 1978) and human breast carcinomas (Allegra *et al.*, 1980; Namer *et al.*, 1980).

It is still not clear, however, whether relapse following regression is due to regrowth of a hormone-independent population. For this reason we have carried out sequential biopsies of accessible skin metastases in patients with breast cancer, and have determined their RE content before and during therapy, and at the time of relapse.

### PATIENTS AND METHODS

*Patients.*—Twenty-six patients (ages 34–94; mean age 67) with skin metastases, extensive local disease or local recurrent breast carcinoma had skin biopsies performed before receiving endocrine therapy, and 2–3 months later, while receiving endocrine

Address for correspondence: Dr R. C. Coombes, Ludwig Institute for Cancer Research (London Branch), Royal Marsden Hospital, Sutton, Surrey SM2 5PX, U.K.

therapy. In addition, a group of 13 patients who had relapsed following a previous response to endocrine therapy, had skin biopsies performed. Biopsies taken at relapse were obtained when the treatment was stopped (3 patients) or 2–6 weeks after therapy was discontinued in the remaining patients. Ten of these patients then received further endocrine therapy.

About 0·5 g tissue was removed under lignocaine local anaesthesia and 0·4 g frozen in liquid $N_2$ and stored for subsequent RE and progesterone receptor (RP) estimations. The remaining 0·1 g was examined histologically after fixation in the standard fashion.

*Histology*.—Histological examination was carried out on 50 skin nodules from 25 patients, where at least 2 skin nodule biopsies (*i.e.* pre- and on treatment) were available. As the cellularity varied from patient to patient and, of course, between the pairs of samples from the same patient, a quantitative scale was devised: +, small number of single cells scattered in the stroma; + + +, confluent sheets of malignant cells occupying most of section. Anything between these two extremes was scored as + +. All sections were routinely stained with haematoxylin and eosin. Some sections were additionally stained for epithelial membrane antigen (EMA), (Sloane & Ormerod, 1981) with the indirect immunoperoxidase method when identification of malignant cells was uncertain. The histological evaluation was completed without any knowledge of the tissue RE content.

*Serum hormone assays*.—Serum samples were taken at the same time as the biopsies. Oestradiol, testosterone, follicle-stimulating hormone (FSH) and luteinizing hormone (LH) were measured by radioimmunoassay, using reagents provided by the WHO Matched Reagents scheme and according to WHO recommended procedures (WHO Method Manual, 1981). Sex-hormone-binding-globulin (SHBG) was measured by the 2-tier column method of Iqbal & Johnson (1977).

*Hormone-receptor assays*.—Receptor assays were performed using the dextran-coated-charcoal method to separate "bound" from "free" hormone (McGuire *et al.*, 1975). Binding data were analysed by the method of Scatchard (1949). Oestrogen and progesterone receptor levels are expressed as fmol/mg of cytosol protein and intra-assay variation was 10%. The interassay variation was 15%. A concentration of receptor $>10$ fmol/mg cytosol protein is regarded as positive.

*Endocrine therapy*.—Eight patients were treated with tamoxifen (T), 7 with aminoglutethimide (A) (Smith *et al.*, 1978) and 3 with Danazol (D) (Coombes *et al.*, 1980) and 5 with a combination of all 3 (TAD). One patient was treated with premarin, one by withdrawal of premarin, and one by oophorectomy.

In the group of 13 patients who had skin biopsies following relapse after endocrine therapy, 4 had been receiving tamoxifen, 5 aminoglutethimide, 2 premarin and 2 oophorectomy. One patient received the TAD combination. Endocrine therapy had been stopped 2–6 weeks before the skin biopsy in 11 cases, and 2 patients were still receiving therapy. One patient who had relapsed after response to tamoxifen responded to further treatment with aminoglutethimide, and had a further skin biopsy when she relapsed on this therapy.

*Response criteria*.—Response was assessed according to UICC criteria (Hayward *et al.*, 1977) with clinical measurement and photography of the skin lesions, and by appropriate investigations to determine response, or non-response at other sites of disease.

(i) *Complete response* (CR) was defined as disappearance of all clinical and radiological disease for at least one month.
(ii) *Partial response* (PR) was defined as $>50\%$ reduction in the product of the 2 largest perpendicular diameters of any measurable lesion, in the absence of any new lesions developing elsewhere, or of further progression of known lesions, for at least a month.

### RESULTS

### *Changes in receptor content of skin nodules during therapy* (Tables I and II)

Skin biopsies were obtained before and during treatment in 26 patients, 12 of whom responded to endocrine therapy (Table I) whilst 14 failed to respond (Table II). RE was $\geqslant 10$ fmol/mg cytosol protein in 9/12 (75%) responders and 9/14 (64%) non-responders. Two out of 3 of the responders without detectable RE had received prior tamoxifen. RE content fell in 8/9 responding patients with measurable

TABLE I.—*Receptor content of sequential biopsies in patients responding to endocrine therapy*

| Patient No. | Age (yr) | Therapy* | Pre-treatment biopsy RE (fmol/mg cytosol protein) | Pre-treatment biopsy Pg.R. (fmol/mg cytosol protein) | Biopsy during treatment RE (fmol/mg cytosol protein) | Biopsy during treatment RP (fmol/mg cytosol protein) | Previous therapy |
|---|---|---|---|---|---|---|---|
| 1 | 60 | D | ⩾10 NQ | — | <10 | <10 | T (stopped 4 weeks prior to D) |
| 2 | 71 | T | <10 | — | <10 | <10 | — |
| 3 | 72 | TAD | 541 | 125 | <10 | <10 | — |
| 4 | 54 | A | <10 | <10 | <10 | <10 | T (stopped 4 weeks prior to A) |
| 5 | 80 | TAD | 162 | <10 | <10 | <10 | P (stopped 3 weeks prior to TAD) |
| 6 | 94 | T | 737 | 380 | <10 | <10 | — |
| 7 | 71 | T | 31 | <10 | <10 | <10 | — |
| 8 | 77 | T | 1186 | 62 | <10 | ⩾10 NQ | — |
| 9 | 72 | A | <10 | <10 | <10 | <10 | T (stopped 4 weeks prior to A) |
| 10 | 72 | A | 19 | <10 | 22 | <10 | D (stopped 3 weeks prior to A) |
| 11 | 70 | P withdrawal | 271 | <10 | <10 | <10 | P (stopped 3 weeks prior to first biopsy) |
| 12 | 54 | P | 150 | <10 | <10 | <10 | A (stopped 16 weeks prior to P) |
| Means | | | 260·2† | 60·2† | 12† | 5·4† | |
| Number showing fall | | | | | 8 | 2 | |
| Number showing rise | | | | | 0 | 0 | |

* T—Tamoxifen; A—Aminoglutethimide; D—Danazol; P—Premarin; RE—Oestrogen receptor; RP—Progesterone receptor; NQ—Non quantifiable;
† <10 taken as 5 fmol/mg protein and ⩾10 taken as 10 fmol/mg protein in calculation of means.

TABLE II.—*Receptor content of sequential biopsies in patients not responding to endocrine therapy*

| Patient no. | Age (yr) | Therapy | Pre-treatment biopsy RE (fmol/mg cytosol protein) | Pre-treatment biopsy RP (fmol/mg cytosol protein) | Biopsy during treatment RE (fmol/mg cytosol protein) | Biopsy during treatment RP (fmol/mg cytosol protein) | Previous therapy |
|---|---|---|---|---|---|---|---|
| 1 | 67 | T | 133 | — | <10 | <10 | — |
| 2 | 76 | T | 410 | — | <10 | <10 | — |
| 3 | 34 | Ooph. | 24 | — | 19 | <10 | — |
| 4 | 71 | TAD | 800 | <10 | 26 | <10 | — |
| 5 | 74 | T | <10 | <10 | <10 | — | — |
| 6 | 72 | D | 18 | <10 | 19 | <10 | — |
| 7 | 72 | TAD | <10 | <10 | <10 | <10 | — |
| 8 | 79 | TAD | 247 | <10 | <10 | <10 | — |
| 9 | 44 | A | 91 | — | 141 | <10 | — |
| 10 | 61 | D | <10 | <10 | <10 | <10 | — |
| 11 | 54 | A | <10 | <10 | <10 | <10 | — |
| 12 | 56 | A | <10 | <10 | <10 | <10 | T (stopped 4 weeks before 1st biopsy) |
| 13 | 83 | T | 376 | 97 | 195 | 37 | — |
| 14 | 52 | A | 48 | <10 | <10 | <10 | T (stopped 4 weeks before 1st biopsy) |
| Mean | | | 155·1 | 14·2 | 31·8 | 7·5 | |
| Number showing fall | | | | | 6 | 1 | |
| Number showing rise | | | | | 6 | 0 | |

receptor. The receptor value fell from a mean of 260·2 fmol/mg (range <10–1186 to 12 fmol/mg (range <10–22) cytosol protein.

In the non-responders, 6/9 patients with measurable RE showed a fall in concentration, and the RE value fell from a mean of 155·1 fmol/mg (range <10 to 800 fmol/mg) to 31·8 fmol/mg (range <10–195 cytosol protein.

Tamoxifen is known to occupy receptor sites, and can therefore lead to negative results. Of those patients who had not received tamoxifen, 3/4 responders and

TABLE III.—*Receptor content of skin metastases from patients who have relapsed after a previous response to endocrine therapy*

| Patient | Age (yr) | Previous therapy | Receptor content of recurrent tumour RE | RP | Subsequent endocrine therapy | Outcome |
|---|---|---|---|---|---|---|
| 1 | 60 | T | <10 | <10 | D | PR |
| 2 | 54 | T | <10 | <10 | A | PR |
| * | | A | 23 | — | D | PD |
| 3 | 80 | P | 162 | <10 | TAD | PD |
| 4 | 72 | T | <10 | <10 | A | PR |
| 5 | 56 | Ooph | 10 | <10 | — | — |
| 6 | 70 | P | 271 | <10 | P. withdrawal | PR |
| 7 | 54 | A | 150 | <10 | P | PR |
| 8 | 52 | T | 48 | <10 | A | PD |
| 9 | 47 | Ooph | 76 | — | T | PD |
| 10 | 65 | A | 167 | — | T | Not assessed |
| 11 | 66 | A | 106 | — | — | — |
| 12 | 59 | TAD | 102 | — | — | — |
| 13 | 82 | A | 84 | — | Norethisterone acetate | NC |

* Relapse after 2nd course of endocrine therapy.

1/4 non-responders showed a fall in RE Prior tamoxifen therapy appeared to give rise to negative results for up to 4 weeks after therapy was stopped. However, this did not appear to prevent patients from responding a second time, since 2 patients with undetectable RE following tamoxifen both responded to subsequent endocrine therapy started shortly after the biopsy was taken (patients 4 and 9, Table I).

*Receptor content of recurrent skin metastases after response to endocrine therapy* (Table III)

Skin biopsies were obtained from 13 patients when they relapsed after responding to a variety of endocrine agents, with one patient being studied on 2 separate occasions. Six of these patients have been included in the responders, and 1 in the non-responders mentioned above. On relapse, 10/14 patients' skin metastases contained measurable RE. One patient, who had been receiving tamoxifen, and whose skin biopsy was $RE^-$ at relapse, responded to further therapy with aminoglutethimide. When she relapsed on this treatment, a repeat skin biopsy was $RE^+$ (patient 12; Table III).

None of the regrowing tumour samples contained measurable progesterone receptor (RP).

*Histology*

Cellularity of skin nodules, obtained from previously untreated patients, appeared to correlate with RE content (Fig.) All 5 samples graded as + and + + con-

FIGURE.—Pre-treatment values for oestrogen receptor (RE) from biopsies from skin metastases, related to the tumour cellularity (graded as +, + + or + + +; see text for definitions). No patient had received tamoxifen before biopsy.

TABLE IV.—*Means ± s.e. of Testosterone, Luteinizing hormone (LH), Follicle-stimulating hormone (FSH), Oestradiol and Sex hormone binding globulin (SHBG) in responding and non-responding patients. Serum samples taken at times of skin biopsies*

| | Responders ($n = 12$) | | Non-responders ($n = 14$) | |
|---|---|---|---|---|
| | Pre-treatment | Post-treatment | Pre-treatment | Post-treatment |
| Testosterone (nmol/l) | 1·85 ± 0·31 | 1·91 ± 0·76 | 2·14 ± 0·53 | 2·46 ± 0·62 |
| LH (u/l) | 24·67 ± 1·56 | 20·50 ± 4·69 | 31·00 ± 9·87 | 34·71 ± 8·99 |
| FSH (u/l) | 45·67 ± 5·97 | 32·33 ± 6·01 | 41·14 ± 8·83 | 51·00 ± 9·79 |
| Oestradiol (pmol/l) | 100·83 ± 10·44 | 115·83 ± 27·66 | 110·71 ± 11·27 | 109·29 ± 19·28 |
| SHGB (nmol DHT bound/l) | 92·00 ± 6·95 | 100·83 ± 18·58 | 72·29 ± 10·83 | 66·43 ± 8·87 |

tained < 50 fmol/mg cytosol protein, in contrast to the more cellular samples. (+ + +).

Concerning the changes of cellularity during therapy, irrespective of type of endocrine treatment, 8/12 responders showed a decrease whereas of 3/10 non-responders showed an increase, 4 showed unchanged cellularity, and 3 showed a decrease.

*Hormone estimations*

Mean values, and standard errors of the means for oestradiol, testosterone, follicle stimulating hormone (FSH), luteinizing hormone (LH) and sex hormone binding globulin (SHBG) taken at the same times as the biopsies from the 26 patients in Tables I and II are shown in Table IV. There was no significant difference between the pre- and post-treatment values for any of these hormones, and no correlation between any individual hormone concentration and RE content.

### DISCUSSION

Our results indicate that the tumour RE content is often lowered by successful endocrine therapy, but rises when the tumours regrow. The initial fall in RE content confirms the findings of other investigators (Allegra *et al.*, 1980, Kiang & Kennedy, 1977; Webster *et al.*, 1978). These changes in RE content appear to parallel the tumour cellularity of the biopsy material; therefore our observed fall in RE content may be related to the tumour-cell content of the specimen. The fall in RE content did not correlate with changes in serum oestradiol or any other hormone measured, indicating that changes in peripheral hormone concentration induced by endocrine therapy are not the cause of changes in RE.

Our finding that 10/14 regrowing tumour samples had significant RE indicates that relapse following regression is not usually due to regrowth of a residual $RE^-$ population of tumour cells. We are not certain that all these tumours were $RE^+$ at the start of treatment, but it is widely accepted that only 4–6% of $RE^-$ tumours respond to therapy (McGuire *et al.*, 1978) so these tumours were likely to have been initially $RE^+$.

An alternative explanation is that endocrine therapy, for example aminoglutethimide, is no longer capable of suppressing endogenous hormone synthesis, thus allowing endocrine-sensitive tumour cells to regrow. Studies at this institute, however, have shown that patients who respond to aminoglutethimide show adequate steroid suppression, even at relapse (Coombes *et al.*, 1981).

Many of the patients in the study were receiving tamoxifen, or a combination. Tamoxifen is known to bind RE, and translocate it into the nucleus (Sutherland & Murphy, 1980) and this may explain why many of these patients became $RE^-$ on therapy. Tamoxifen is known to have a long half-life, which has been calculated as ~5·3 days (Wilkinson *et al.*, 1980) accounting for the prolonged RE negativity of the tumours biopsied 2–4 weeks after stopping tamoxifen. However, some of the patients not receiving tamoxifen also showed a marked fall in RE.

Since patients often relapse with RE+ tumours, the biochemical changes that have occurred to enable these tumours to regrow in an unfavourable endocrine environment are not clear. A better understanding of these mechanisms could have therapeutic implications for maintenance of endocrine remission.

### REFERENCES

ALLEGRA, J. C., BARLOCK, A., HUFF, K. K. & LIPPMANN, M. E. (1980) Changes in multiple or sequential oestrogen receptor determinations. *Cancer*, **45**, 792.

ARAFAH, B. M., GULLINO, P. M., MANNI, A. & PEARSON, O. H. (1980) Effect of ovariectomy on hormone receptors and growth of N-nitroso-methyl-urea-induced mammary tumours in the rat. *Cancer Res.*, **40**, 4628.

BODWIN, J. S., CLAIR, T. & CHO-CHUNG, Y. S. (1978) Inverse relation between oestrogen receptors and cyclic adenosine 3:5 monophosphate-binding proteins in hormone-dependent mammary tumour regression due to dibutyryl cyclic adenosine 3:5 monophosphate treatment or ovariectomy. *Cancer Res.*, **38**, 3410.

CHO-CHUNG, Y. S., BODWIN, J. S. & CLAIR, T. (1978) Cyclic-AMP binding proteins: Inverse relationship with oestrogen receptors in hormone dependent mammary tumour regression. *Eur. J. Biochem.*, **86**, 51.

COOMBES, R. C., DEARNALEY, D., HUMPHREYS, J. & 5 others (1980) Danazol treatment in advanced breast cancer. *Cancer Treat. Rep.*, **64**, 1073.

COOMBES, R. C., JARMAN, M., HARLAND, S. & 7 others (1981) Aminoglutethimide: Metabolism and effects on steroid synthesis *in vivo*. *J. Endocrinol*, **87**, 31.

HAYWARD, J. L., CARBONE, P. P., HEUSON, J.-C., KUMAOKA, S., SEGALOFF, A. & RUBENS, R. D. (1977) Assessment of response to therapy in advanced breast cancer. *Cancer*, **39**, 1289.

IQBAL, M. J. & JOHNSON, M. W. (1977) Study of steroid-protein binding by a novel "2-tier" column employing cibacron blue F3G-A-Sepharose 4B. I. Sex hormone binding globulin. *J. Steroid Biochem.*, **8**, 977.

KIANG, D. T. & KENNEDY, B. J. (1977) Factors affecting oestrogen receptors in breast cancer. *Cancer*, **40**, 1571.

MCGUIRE, W. L. (1978) Hormone receptors: Their role in predicting prognosis and response to endocrine therapy. *Sem. Oncol.*, **5**, 428.

MCGUIRE, W. L., CARBONE, P. P., SEARS, M. E. & ESCHER, G. C. (1975) In *Oestrogen Receptors in Human Breast Cancer* Ed. McGuire *et al.* New York: Raven Press. p. 1.

NAMER, M., LALANNE, C. & BAULIEU, E. E. (1980) Increase of progesterone receptor by tamoxifen as a hormonal challenge test in breast cancer. *Cancer Res.*, **40**, 1750.

SCATCHARD, G. (1949) The attractions of protein for small molecules and ions. *Ann. N.Y. Acad. Sci.*, **51**, 660.

SLOANE, J. P. & OMEROD, M. G. (1981) Distribution of epithelial membrane antigen in normal and neoplastic tissues and its value in diagnostic tumour pathology. *Cancer.*, **47**, 1048.

SMITH, I. E., FITZHARRIS, B. M., MCKINNA, J. A. & 6 others (1978) Aminoglutethimide in the treatment of metastatic breast cancer. *Lancet*, ii, 646.

SUTHERLAND, R. L. & MURPHY, L. C. (1980) The binding of tamoxifen to human mammary carcinoma cytosol. *Eur. J. Cancer*, **16**, 1141.

WEBSTER, D. J. T., BROWN, D. G. & MINTON, J. P. (1978) Oestrogen receptor levels in multiple biopsies from patients with breast cancer. *Am. J. Surg.*, **136**, 337.

WHO (1981) Programme for the provision of matched assay reagents for the radioimmunoassay of hormones in reproductive physiology. *Method Manual.* 5th Edn.

WILKINSON, P., RIBEIRO, G., ADAM, H. & PATTERSON, J. (1980) Clinical pharmacology of tamoxifen and N. desmethyltamoxifen in patientes with advanced breast cancer. *Cancer Chemother. Pharmacol.*, **5**, 109.